# A case of infantile spasms with three possibly pathogenic de novo missense variants in *NF1* and *GABBR1*

**DOI:** 10.1038/s41439-023-00256-7

**Published:** 2023-11-22

**Authors:** Kazuki Watanabe, Kazuo Kubota, Mitsuko Nakashima, Hirotomo Saitsu

**Affiliations:** 1 Department of Biochemistry, Hamamatsu University School of Medicine, Hamamatsu, Japan; 2 Department of Pediatrics, Gifu University Graduate School of Medicine, Gifu, Japan; 3 Division of Clinical Genetics, Gifu University Hospital, Gifu, Japan

**Keywords:** Epilepsy, Neurodevelopmental disorders

## Abstract

Neurofibromatosis type 1 (NF1) is one of the most common hereditary neurocutaneous disorders. Here, we report a unique case of a patient with typical NF1 findings and infantile spasms who had three possibly pathogenic de novo variants, c.3586C>T, p.(Leu1196Phe) and c.3590C>T, p.(Ala1197Val) in *NF1* located in cis and c.1042G>C, p.(Ala348Pro) in *GABBR1*. This study contributes to our understanding of the effect of two cis variants on NF1 phenotypes and *GABBR1*-related neuropsychiatric disorders.

Neurofibromatosis type 1 (NF1, OMIM# 162200) is one of the most common hereditary neurocutaneous disorders, with an estimated birth prevalence of 1 in 2,000–3,000 individuals^1^ caused by heterogeneous variants in neurofibromin 1 (*NF1*). Most individuals with NF1 exhibit characteristic cutaneous phenotypes, including hyperpigmented nevus (café au lait spots and axillary freckling) and fibromatous tumors of the skin. Seizure is one of the neurological disorders common in NF1, with a recent study revealing that the prevalence of epilepsy in NF1 patients is approximately 5.4%^2^. Infantile spasms (ISs) are a common type of epilepsy in infancy, with a prevalence of 2–5 per 10,000 live births^3^, and have a heterogeneous genetic background. However, IS is rarely observed in NF1 individuals, with a prevalence of only 0.62–0.90%^4^.

In a recent study, four heterozygous missense variants in the gamma-aminobutyric acid type B receptor subunit 1 (*GABBR1*) gene were associated with motor and/or language delay and epilepsy^5^. *GABBR1* encodes a gamma-aminobutyric acid (GABA)_B1_ subunit, which forms a heterodimeric G-protein coupled receptor with GABA_B2_. GABA is a major inhibitory neurotransmitter in the central nervous system, and pathogenic variants in genes involved in the GABA metabolic pathway cause epilepsy^6^. One patient with a *GABBR1* variant exhibited epilepsy. However, the phenotypic spectrum of *GABBR1*-related disorders remains unclear.

Here, we report a unique case of an NF1 patient with ISs, possessing three possibly pathogenic de novo variants, namely, two missense variants in *NF1* and one missense variant in *GABBR1*. We review the literature and discuss the phenotypic characteristics of our patient and the contribution of each genetic variant to the pathology of NF1 with IS.

The patient was a Japanese girl who was the third child of unrelated healthy parents. She was born at 37 weeks gestation by cesarean section without asphyxia after an uneventful pregnancy. Her birth weight, body length, and occipitofrontal circumference (OFC) were 3072 g (1.3 standard deviations (SD)), 48 cm (0.11 SD), and 33.5 cm (0.45 SD), respectively. She exhibited ears with overfolded helices that spontaneously improved and left developmental dysplasia of the hip (DDH), which was treated with a Pavlik harness. Her mother and the elder brother of her maternal grandfather had DDH, but there was no family history of neurodevelopmental disorders. At 5 months of age, she started to experience seizures with spasms during crying, and the clusters of epileptic spasms gradually increased. She was able to smile and acquired head control at 3 months of age, but developmental regression, such as lost smiling, was observed. Electroencephalography (EEG) at 5 months of age showed multifocal sharp waves and spike and wave complexes, which are characteristic of hypsarrhythmia (Fig. 1A). Brain magnetic resonance imaging (MRI) at 5 months of age was normal (Fig. 1B). Her seizures disappeared with valproic acid and adrenocorticotropic hormone (ACTH) injection, and the EEG was normalized. Brain computed tomography at 7 months of age showed cerebral lobe shrinkage and enlargement of the subdural space, which is associated with ACTH injections (Fig. 1C), and ACTH injections were discontinued. Her developmental milestones were slightly delayed; she rolled over at 10 months, walked with support at 1 year and 1 month, crawled at 1 year and 3 months, said words at 1 year and 6 months, walked independently at 1 year and 7 months and spoke some sentences at 3 years and 3 months. At the final examination at 3 years and 6 months, her weight, body height, and OFC were 15.2 kg (1.80 SD), 92.1 cm (‒0.74 SD), and 49.3 cm (0.29 SD), respectively. There were seven café au lait spots >5 mm on her chest and back and four or more café au lait spots <5 mm. No freckling, neurofibromas, Lisch nodules, choroidal abnormalities or distinctive osseous lesions were observed. The developmental quotient measured by the Kyoto Scale of Psychological Development was 53.

**Fig. 1:**
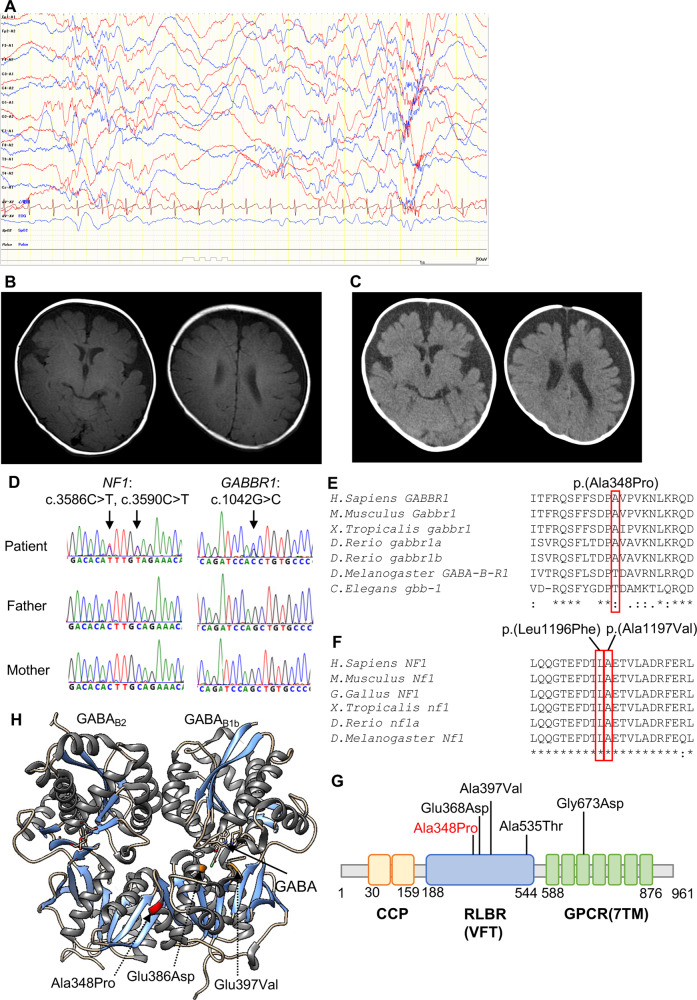
A patient with de novo missense variants in *NF1* and *GABBR1*. **A** EEG at 5 months showed hypsarrhythmia-like discharges. **B**, **C** Brain MRI at 5 months was normal, but brain CT at 6 months after ACTH treatment showed brain atrophy. **D** Electropherograms for each examined individual. **E**, **F** Alignment of the multispecies protein sequences of *NF1* and *GABBR1*. **G** Schematic diagram of *GABBR1* protein structure, together with reported and identified variants. The variant found in this study is indicated by red letters. The yellow, green, and blue boxes indicate the Sushi/SCR/CCP domain, receptor ligand-binding region (VFTD), GPCR family 3, and C-terminal domain (7TMD), respectively. These domains were annotated using InterPro. **H** Heterodimeric extracellular GABA receptor VFT in the active state and GABA (PDB ID: 4MS4). The positions of the previously reported protein variants are shown in orange, and the positions of protein variants seen in this case are shown in red.

Case-only exome sequencing (ES) for the patient detected three possibly pathogenic variants: c.3586C>T, p.(Leu1196Phe) and c.3590C>T, p.(Ala1197Val) in *NF1* (NM_001042492.3) and c.1042G>C, p.(Ala348Pro) in *GABBR1* (NM_001470.4). The Integrative Genomics Viewer showed that the neighboring two *NF1* variants originated from the same allele (Supplemental Fig. S1). Sanger sequencing confirmed that all variants were de novo (Fig. 1D). According to the American College of Medical Genetics and Genomics standards and guidelines, the c.3586C>T, p.(Leu1196Phe) and c.3590C >T, p.(Ala1197Val) variants in *NF1* were classified as “pathogenic” (PS1, PS2, PM2, PP3) and “likely pathogenic” (PS2, PM2, PP3), respectively. The c.1042G>C, p.(Ala348Pro) variant in *GABBR1* was classified as “likely pathogenic” (PS2, PM1, PM2, PP3). Detailed analysis methods and the pathological evaluation of each variant are described in the Supplementary Methods. According to the revised diagnostic criteria for neurofibromatosis type 1^7^, our patient was diagnosed with NF1.

Here, we present a rare case of three de novo variants in two genes, *NF1* and *GABBR1*. The patient had ISs, which are a rare symptom in NF1 patients. To clarify the phenotypic characteristics of our patient, we evaluated two previously reported patients with the same *NF1* variant (c.3586C>T, p.(Leu1196Phe))^8,9^, 10 NF1 patients presenting with IS^4^, and 4 patients with *GABBR1* variants^5^ (Table 1). First, we compared the clinical findings between our patient and two previous patients with the same variant (c.3586C>T, p.(Leu1196Phe))^8,9^. No obvious differences were observed in their clinical characteristics, except for IS. Epilepsy is a relatively rare phenotype in NF1, and a recent systematic review reported an estimated lifetime prevalence of epilepsy in NF1 patients of approximately 5.4%, with lower values (<4%) in pediatric patients^2^. IS accounts for approximately 10% of NF1 epilepsy cases, 32% of which show some structural brain lesions, including brain tumors and cortical abnormalities^2^. Comparing 10 NF1 patients with IS^4^, no significant differences were observed in terms of developmental abnormalities, physical findings, or response to treatment. However, six patients showed macrocephaly and brain abnormalities were observed in four of these six cases, suggesting that they may have some cortical migration disorders.

**Table 1:** Clinical summary of cases with NF1 and *GABBR1*-related neuropsychiatric disorders.

Case	*GABBR1* cases (4 patients)^5^	NF1 with IS (10 patients)^4^	Two patients with the same *NF1* variant^8,9^	Our patient
*GABBR1* (NM_001470.4)	Missense 4	−	−	c.1042G>C, p.(Ala348Pro)
*NF1* (NM_001042492.3)	–	Germ-line mutations 9/10 (details unknown)	c.3586C>T, p.(Leu1196Phe)	c.3586C>T, p.(Leu1196Phe) c.3590C>T, p.(Ala1197Val)
Sex	M1, F3	M 6, F 4	M 2, F 0	F
Age	Mean 9.25 y (2.5–17)	Mean 12.9 y (2–26)	Mean 7.9 y (5.3–10.5)	3y4m
Head circumference	Macrocephaly 1/4	Macrocepahly 6/10	N.A.	Normal range (0.29 SD)
Height	Normal range 4/4	Short statue 3/10	Short stature 2/2	Normal range (−0.74 SD)
Weight (kg)	Normal range 4/4	N.A.	N.A.	Normal range (1.80 SD)
Motor delay	4/4	N.A.	1/1 (1 N.A.)	+
Hypotonia	2/4	N.A.	1/1 (1 N.A.)	−
Speech delay or abnormalities	2/4	N.A.	1/1 (1 N.A.)	+
Intellectual disability	2/3	DD or ID 7/10	N.A.	+
Epilepsy	1/4 (GTS, absence)	IS 10/10	0/2	IS
Seizure onset (months)	N.A.	mean 6.3 (3-9)	N.A.	5
Facial abnormality	Facial abnormality 1	N.A.	Facial abnormality 1 (1 N.A.)	−
NF1 feature				
Café au lait spot	0/4	10/10	2/2	+
Freckling	0/4	9/10	1/2	−
Lisch nodules	0/4	7/10	0/1 (1 N.A.)	−
Neurofibromas	0/4	9/10	0/2	−
EEG	N.A.	Hyps 8/10, Modified 2/10	N.A.	Modified
Neuroimaging	N.A.	Abnormal (UBO, ScBF, EV) 6/10	Normal 1/1 (1 N.A.)	N
Steroid response	N.A.	Good 5/10, Poor 5/10	N.A.	Good

*DD* developmental delay, *EV* enlarged ventricles, *F* female, *GTS* generalized tonic seizure, *Hyps* hypsarrhythmia, *IS* infantile spasm, *M* male, *Modified* modified hypsarrhythmia, *ID* Intellectual disability, *N* normal, *N.A.* not available, *UBO* high-signal lesions on T2-weighted images, *ScBF* subcortical bright foci.

Our patient had two closely spaced de novo variants in *NF1* on the cis allele. It has been reported that multiple mutations occur simultaneously in a narrow region at a significantly higher rate than expected^10^. This simultaneous occurrence of multiple variants in close proximity is termed closely spaced multiple mutations (CSMMs). It has been postulated that CSMMs arise from transient hypermutability, which is a nonheritable mutagenic condition assumed to be caused by dysregulation of the maintenance of replication fidelity and nucleotide pool imbalance. However, the detailed mechanism remains unclear. It has also been suggested that noncanonical (non-B) DNA structures, with alternative conformations differing from the canonical right-handed DNA helix, which has 10 bp per turn, may promote the generation of CSMMs. We searched for the non-B DNA structures in *NF1* sequences using the non-B DNA Motif Search Tool^11^ but found no non-B DNA structures around the identified variants. There have been reports of three families with NF1 thought to be caused by CSMMs^12,13^. Previous studies suggest that double de novo variants do not contribute to the severity of the clinical phenotypes of NF1 patients. Our patient showed clinical findings similar to those of patients with single de novo variants, suggesting that two de novo variants on cis alleles may have a minor effect on the severity of clinical findings.

To date, four patients with de novo *GABBR1* variants have been reported^5^. All of them had de novo missense variants and presented with developmental delay and varying degrees of intellectual disability, and only one patient presented with epilepsy. We could not find obvious differences between the neurological phenotypic spectrum of NF1 and that of *GABBR1*-related disorder because of the small number of *GABBR1* cases. Although we assumed that the *GABBR1* variant might have additive effects on our patient’s symptoms, it was difficult to distinguish the involvement of the pathogenic variants in the two genes from their clinical manifestations.

The GABA_B_ receptor is a heterodimer composed of two subunits, GABA_B1_ and GABA_B2_, which consist of an extracellular Venus flytrap (VFT) domain and a heptahedral transmembrane (7TM) domain, respectively (Fig. 1G). The orthosteric binding site of the GABA_B_ receptor is located in the VFT of the GABA_B1_ subunit, while the 7TM domain of the GABA_B2_ subunit is mainly responsible for recruiting G proteins, in addition to hosting an allosteric binding site^14^. The previously reported variants p.Glu368Asp and p.Ala397Val are located near the orthosteric binding site, and functional analysis has shown that p.Glu368Asp reduces the potency and efficacy of GABA at the receptor, while p.Ala397Val decreases the efficacy of GABA at the receptor^5^. In contrast, the p.Ala348Pro variant found in our patient was located in the same VFT domain but away from the orthosteric binding site (Fig. 1H). Our variant was located near the binding sites of GABAB1 and GABAB2^14^ and may affect the heterodimerization of the GABA_B_ receptor, impairing agonist-dependent Gi protein activation. Although the c.1042G>C, p.(Ala348Pro) variant is classified as “likely pathogenic”, its biological influence on the pathology is uncertain. Therefore, we consider that the significance of this variant is currently unknown. Further accumulation of cases and functional analysis are needed to support the pathogenicity of this variant.

In conclusion, we present a patient with two de novo CSMM variants in *NF1* and a de novo *GABBR1* variant. Although it was difficult to clarify the involvement of each variant in our patient’s neurological pathology, this study contributes to our understanding of the effect of CSMMs on NF1 phenotypes and *GABBR1*-related neuropsychiatric disorders.

## HGV Database

The relevant data from this Data Report are hosted at the Human Genome Variation Database at 10.6084/m9.figshare.hgv.3337; 10.6084/m9.figshare.hgv.3340; 10.6084/m9.figshare.hgv.3343.

### Supplementary information

Supplementary methods, Figure S1, Table S1

## Data Availability

The data that support the findings of this study are available from the corresponding author upon reasonable request.
