# Supplementary material for: A case of infantile spasms with three possibly pathogenic de novo missense variants in NF1 and GABBR1
Source: Hum Genome Var. 2023 Nov 22;10:30. doi: 10.1038/s41439-023-00256-7 (PMC10665374; doi:10.1038/s41439-023-00256-7)
Supplement: Supplementary file 1 — Supplementary methods, Figure S1, Table S1 [file 41439_2023_256_MOESM1_ESM.docx]

**Supplementary methods**

This study was approved by the Institutional Review Board Committee of Hamamatsu University School of Medicine and Gifu University. Written informed consent for genetic analysis and publication of results was obtained from the family.

The patient’s DNA was captured using an xGen Exome Research Panel kit (IDT, Coralville IA) and sequenced on a NextSeq500 (Illumina, San Diego, CA) with 75-bp paired-end reads. Local realignment and base quality recalibration were performed using GATK (Version 4.1.9.0), and variants were called using GATK HaplotypeCaller. The final variants were annotated using Annovar to predict the functional impact of the coding variants and to assess allele frequency using the following databases: an in-house database of 82 control exomes, the human genetic variation database^1^, the ToMMo 38KJPN-SNV/INDEL Genotype Frequency Panel^2^, and the Genome Aggregation Database (gnomAD)^3^. We focused on rare variants with minor allele frequencies <1% in the above four databases. The damaging prediction was performed using SIFT^4^, Polyphen-2^5^, MutationTaster^6^, and CADD^7^ programs. Candidate variants were confirmed by Sanger sequencing using an ABI 3130xl Genetic Analyzer (Applied Biosystems, Foster City, CA). The biological parentage was confirmed by analyzing 10 microsatellite markers (data not shown).

We searched for the candidate variants in the genes associated with epilepsy using WES data and found three possible pathogenic variants: c.3586C>T, p.(Leu1196Phe) and c.3590C>T, p.(Ala1197Val) in *NF1* (NM_001042492.3), and c.1042G>C, p.(Ala348Pro) in *GABBR1* (NM_001470.4). The c.3586C>T, p.(Leu1196Phe) variant was previously reported as pathogenic^8, 9^, while the other two variants were novel. Integrative Genomics Viewer showed that the neighboring two *NF1* variants originated from the same allele (Supplemental Figure S1). Sanger sequencing confirmed that all variants were *de novo* (Figure D). All variants were absent from the public databases, including the Genome Aggregation Database (gnomAD) v3.1.2^3^, ToMMo 38KJPN Allele Frequency Panel^2^, and 82 in-house Japanese exome control data. All three variants were evolutionarily highly conserved (Figure E and H) and predicted to be deleterious by multiple pathogenicity prediction tools (Supplemental Table S1). Based on American College of Medical Genetics and Genomics standards and guidelines, the c.3586C>T, p.(Leu1196Phe) and c.3590C>T, p.(Ala1197Val) variants in *NF1* were classified as “pathogenic” (PS1, PS2, PM2, PP3) and “likely pathogenic” (PS2, PM2, PP3), respectively. The c.1042G>C, p.(Ala348Pro) variant in *GABBR1* was classified as “likely pathogenic” (PS2, PM1, PM2, PP3).

**Supplementary references**.

1. Human genetic variation database. <http://www.hgvd.genome.med.kyoto-u.ac.jp/>. Accessed 17 August 2023.

2. ToMMo 38KJPN-SNV/INDEL Genotype Frequency Panel (v20220929). <https://jmorp.megabank.tohoku.ac.jp/downloads/tommo-38kjpn-20220929-gf_snvindelall>. Accessed 17 August 2023.

3. gnomAD . Available from: <https://gnomad.broadinstitute.org/>. Accessed 17 August 2023.

4. Ng PC, Henikoff S. SIFT: Predicting amino acid changes that affect protein function. Nucleic Acids Res. 2003; 31: 3812-3814.

5. Adzhubei IA, Schmidt S, Peshkin L, Ramensky VE, Gerasimova A, Bork P, et al. A method and server for predicting damaging missense mutations. Nat Methods. 2010; 7: 248-249.

6. Schwarz JM, Cooper DN, Schuelke M, Seelow D. MutationTaster2: mutation prediction for the deep-sequencing age. Nat Methods. 2014; 11: 361-362.

7. Kircher M, Witten DM, Jain P, O'Roak BJ, Cooper GM, Shendure J. A general framework for estimating the relative pathogenicity of human genetic variants. Nat Genet. 2014; 46: 310-315.

8. Croonen EA, Yntema HG, van Minkelen R, van den Ouweland AMW, van der Burgt I. Patient with a neurofibromatosis type 1 mutation but a clinical diagnosis of Noonan syndrome. Clin Dysmorphol. 2012; 21: 212-214.

9. Kang E, Kim YM, Seo GH, Oh A, Yoon HM, Ra YS, et al. Phenotype categorization of neurofibromatosis type I and correlation to NF1 mutation types. J Hum Genet. 2020; 65: 79-89.

**Figure S1. The aligned reads in *NF1* visualized by IGV**


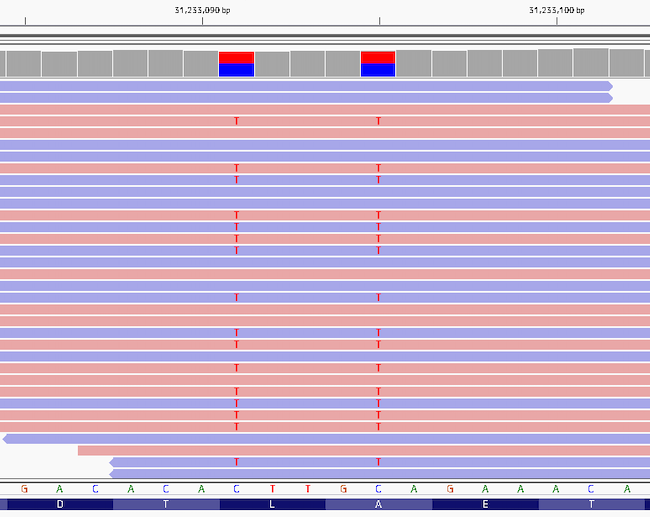


Two variants (c.3586C>T, and c.3590C>T) are present in the same reads.

**Table S1. Candidate variants identified by WES**

| **Chr** | **Gene** | **Variant** | **Origin** | **gnomAD/38KJPN** | **SIFT** | **PP2 HVAR** | **CADD phred** | **M-CAP** | **GERP** | **MutationTaster** |
| --- | --- | --- | --- | --- | --- | --- | --- | --- | --- | --- |
| 17 | *NF1* | NM_001042492.3:c.3586C>T:p.(Leu1196Phe) | *De novo* | −/− | 0.003 | 0.629 | 24.7 | 0.790 | 4.76 | 1 |
| 17 | *NF1* | NM_001042492.3:c.3590C>T:p.(Ala1197Val) | *De novo* | −/− | 0.003 | 0.962 | 26.5 | 0.856 | 5.73 | 1 |
| 6 | *GABBR1* | NM_001470.4:c.1042G>C:p.(Ala348Pro) | *De novo* | −/− | 0.053 | 0.995 | 26.1 | 0.202 | 4.39 | 1 |

SIFT (Sorting Intolerant From Tolerant): https://sift.bii.a-star.edu.sg/, Polyphen-2 Hum Var: http://genetics.bwh.harvard.edu/pph2/, CADD (Combined Annotation–Dependent Depletion): http://cadd.gs.washington.edu/score, M-CAP (Mendelian Clinically Applicable Pathogenicity): http://bejerano.stanford.edu/mcap/index.html, GERP (Genomic Evolutionary Rate Profiling): http://mendel.stanford.edu/SidowLab/downloads/gerp/, MutationTaster: http://www.mutationtaster.org/.
